# Clonal Dynamics of GPI‐Deficient Cells in Patients With Paroxysmal Nocturnal Hemoglobinuria (PNH): A Retrospective Follow‐Up Analysis

**DOI:** 10.1111/ejh.70293

**Published:** 2026-08-21

**Authors:** Sandra M. Frey, Friederike Poppenborg, Ute Schmücker, Nicole Preising, H. Christian Reinhardt, Alexander Röth, Ferras Alashkar

**Affiliations:** ^1^ Department of Hematology and Stem Cell Transplantation, West German Cancer Center University Hospital Essen, University of Duisburg‐Essen Essen Germany; ^2^ Department of Hematology and Central Hematology Laboratory, Inselspital University Hospital Bern, University of Bern Bern Switzerland

**Keywords:** clonal dynamics, FLAER, GPI‐deficient cells, paroxysmal nocturnal hemoglobinuria

## Abstract

This retrospective, single‐center study aimed to characterize clonal dynamics of GPI‐deficient cells in patients with paroxysmal nocturnal hemoglobinuria (PNH) or PNH/aplastic anemia (AA) syndrome using multiparameter flow cytometry including FLAER. Among 108 patients diagnosed and treated at our center, 83 had longitudinal monitoring of GPI‐deficient neutrophils; 53 of these also had data on monocytes and lymphocytes. Median observation time was 29.4 months (range 1.1–162.7 months). Clone size in neutrophils showed a strong correlation with monocytes, but not with lymphocytes. Clonal persistence represented the most frequent individual pattern of clone evolution observed during follow‐up; clonal persistence was observed in 32/83 patients, while 24 patients exhibited clonal expansion (≥ 10%) and 13 showed regression. Expansion frequently involved all three lineages, suggesting ongoing biological mechanisms contributing to clonal selection. Regression was more likely in patients with small initial clones. In six patients undergoing allogeneic stem cell transplantation, complete eradication of PNH clones was achieved. Clonal dynamics were independent of eculizumab treatment; however, regression was observed in some patients treated with ATG and cyclosporine A. These findings support the need for longitudinal clone size monitoring, particularly in neutrophils and monocytes. Further studies are warranted to elucidate factors influencing clone expansion and remission.

**Trial Registration:** 25‐12 634‐BO

## Novelty Statement

1. What is the NEW aspect of your work? This study provides additional real‐world longitudinal data on clonal dynamics across neutrophils, monocytes, and lymphocytes in patients with PNH and AA/PNH using Multiparameter flow cytometry including FLAER.
2. What is the CENTRAL finding of your work? Whereas most PNH clones remained stable over time, a significant clonal expansion and regression occurred in a subset of patients, independent of eculizumab treatment.
3. What is (or could be) the SPECIFIC clinical relevance of your work? Serial monitoring of PNH clone size in neutrophils and monocytes enables more accurate disease assessment and may help guide individualized treatment decisions.

## 1 Introduction

Paroxysmal nocturnal hemoglobinuria (PNH) is an acquired, nonmalignant clonal blood disorder caused by a somatic mutation in a pluripotent hematopoietic stem cell or a hematopoietic progenitor cell. PNH can arise in the context of preexisting bone marrow disorders such as aplastic anemia (AA), myelodysplastic syndrome (MDS), or primary myelofibrosis (PMF), or occur de novo. The PNH clone thereby acquires the capacity for expansion and self‐renewal.

The somatic mutation in PNH typically affects the PIGA gene (phosphatidylinositol glycan anchor biosynthesis class A), located on the X chromosome. The PIGA gene is essential for the biosynthesis of the glycosylphosphatidylinositol (GPI) anchor, and its mutation results in defective synthesis of the GPI anchor resulting in partial or complete absence of GPI‐anchored proteins such as CD59 (membrane inhibitor of reactive lysis, MIRL) and CD55 (decay‐accelerating factor, DAF), leading to partial or complete absence of these proteins. This defect increases the susceptibility of affected cells—particularly erythrocytes—to complement‐mediated intravascular hemolysis. Other characteristic clinical features include thrombophilia, manifesting as venous and arterial thromboses, and cytopenias due to bone marrow failure [1, 2].

Flow cytometry using antibodies directed against GPI‐anchored proteins (GPI‐APs) is the most sensitive method for the diagnosis of PNH and is therefore considered to be the diagnostic gold standard. Quantification of at least two GPI‐AP structures (markers) per cell lineage is recommended to exclude an inherited deficiency of a single GPI‐AP [3, 4]. For white blood cells, this is commonly achieved by combining FLAER with CD24 on neutrophils and FLAER with CD14 on monocytes. For erythrocytes, recommendations differ: ICCS/ESCCA guidelines primarily recommend CD59 analysis because of limitations associated with CD55 and CD58 reagents, whereas the German approach recommends assessment of two GPI‐linked antigens on red blood cells, which is also reflected in INSTAND external quality assessment requirements [5]. Flow cytometry not only identifies GPI‐AP‐deficient cells but also allows quantification of the percentage of deficient cells and detection of subpopulations with varying degrees of deficiency, especially among erythrocytes. PNH Type III cells are erythrocytes with complete GPI‐AP deficiency; Type II cells show partial deficiency, while Type I cells express GPI‐APs normally. For accurate diagnosis, it is recommended to evaluate at least two distinct GPI‐AP structures on two different cell lineages, typically neutrophils and erythrocytes [6, 7, 8, 9]. Monocytes may also be analyzed as an additional leukocyte population for confirmation of PNH clone size. Reticulocyte analysis may provide Supporting Information regarding erythroid clone composition, whereas assessment of lymphocytes and platelets is generally considered optional. FLAER (fluorescently labeled pro‐aerolysin) is a reagent used in multiparameter flow cytometry that binds directly to GPI anchors and therefore enables sensitive detection of GPI‐deficient leukocytes, particularly neutrophils and monocytes [7, 10, 11]. FLAER cannot be used for erythrocytes, as they lack the surface‐bound proteolytic enzymes required to process pro‐aerolysin. It is important to note that flow cytometric analysis of erythrocytes may be confounded by recent red blood cell transfusions, which artificially increase the proportion of cells with a normal expression of CD55 and CD59. Therefore, neutrophil and monocyte clone sizes are generally considered robust indicators of the underlying PNH clone size. In conclusion, erythrocyte‐based analysis may underestimate the actual clone size due to intravascular hemolysis and recent red blood cell transfusions. For this reason, PNH clone size is reported as the highest percentage observed in either neutrophils, monocytes, or erythroid cells [10, 12, 13, 14].

## 2 Patients and Methods

Initially, GPI‐deficient neutrophils were identified using FLAER in 108 patients. Of these, 83 patients (76.9%) had longitudinal data available, allowing evaluation of clonal dynamics in neutrophils over time. Within this group of 83 patients, PNH clones on monocytes and lymphocytes were additionally documented in 53 patients. Among these 83 patients, 54 (65.1%) had classic PNH, 28 (33.7%) had an AA/PNH, and one patient (1.2%) had a history of MDS. The median observation period was 29.4 months (range 1.1–162.7 months) until the final day of observation. Patients were treated according to consensus guidelines for PNH and AA at that time. Flow cytometric analyses were performed using FLAER in combination with lineage‐specific and GPI‐linked markers according to published recommendations for high‐sensitivity PNH testing [7, 10, 11, 15, 16]. Neutrophils were identified using CD15 and CD45 in combination with FLAER and CD24, whereas monocytes were identified using CD64 and/or CD14 in combination with FLAER. Lymphocytes were identified based on their characteristic forward and side scatter properties together with CD45 expression. Gating procedures were performed according to established international recommendations for PNH diagnostics and monitoring [7, 10, 11, 15, 16]. Treatment comprised three main modalities: intravenous administration of the terminal complement inhibitor eculizumab; immunosuppressive therapy with antithymocyte globulin (ATG) and cyclosporine A (CsA); and allogeneic hematopoietic stem cell transplantation (HSCT).

PNH clones were analyzed in peripheral blood by multiparameter flow cytometry including FLAER. For analytical consistency, PNH clone size was defined as the highest proportion of GPI‐deficient cells detected across neutrophil, monocyte, or erythroid lineages. Of note, no formal correlation analysis was performed between PNH clone size and clinical parameters, including fatigue, performance status, transfusion requirement, hemoglobin levels, or laboratory markers of hemolysis.

## 3 Results

The overall initial median clone size of GPI‐deficient neutrophil among the 83 evaluable patients was 62.7% (range 0.1%–99.7%). In a subset of 53 patients, clonal dynamics were also assessed in monocytes and lymphocytes. The initial median clone size of GPI‐deficient monocytes was 51.5% (range 0.1%–98.5%) and 3.2% (range 0.01%–31.8%) in GPI‐deficient lymphocytes. At final follow‐up, the median clone size was 70.9% (range 0%–99.8%) in neutrophil (*n* = 83), 59.5% (range 0%–99.5%) in monocytes (*n* = 53), and 3.81% (range 0%–32.4%) in lymphocytes (*n* = 53) (Figure 1).

**FIGURE 1:**
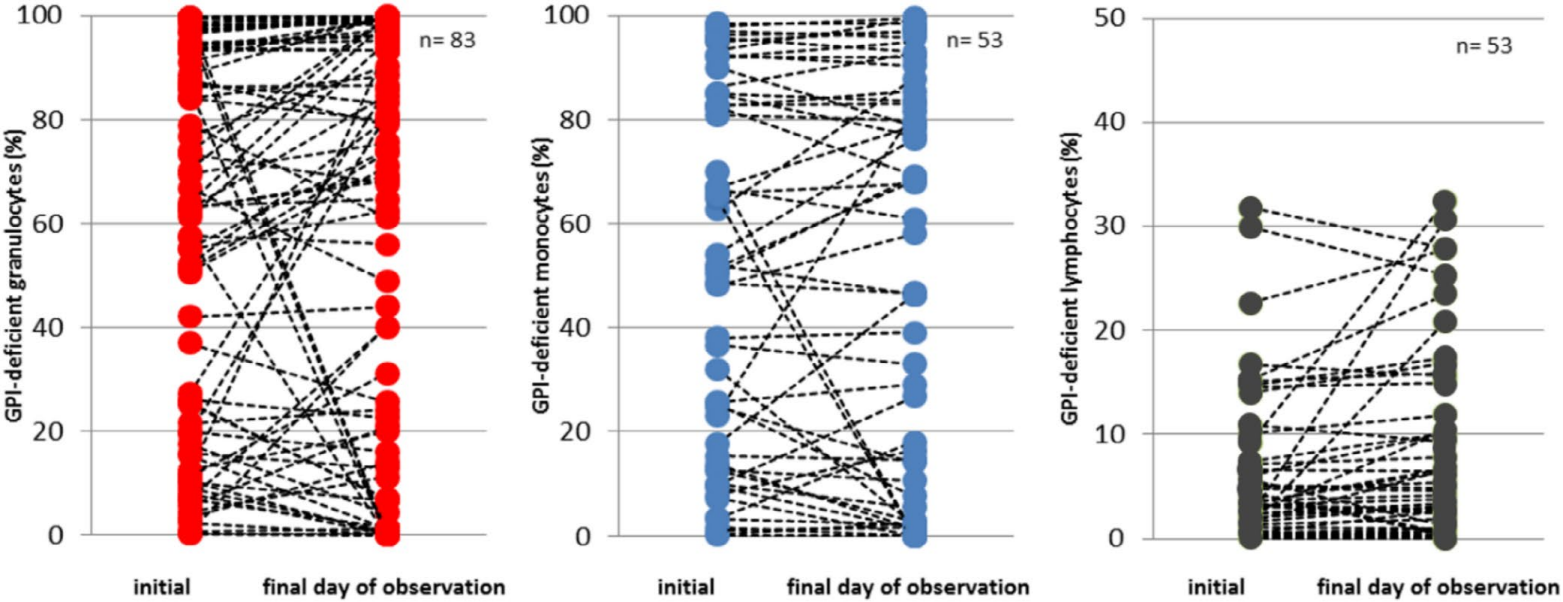
Initial and final median clone size of GPI‐deficient granulocytes, monocytes, and lymphocytes. Red dots granulocytes. Blue dots monocytes. Grey dots lymphocytes.

To assess the relationship between initial clone sizes across the three major cell lineages, GPI‐deficient neutrophils were evaluated in the 53 patients for whom corresponding baseline data on monocytes and lymphocytes were available. In this subgroup, the median initial clone size in neutrophils was 57.6% (range 0.1%–99.7%). Comparison of initial clone sizes in neutrophils and monocytes revealed that in 77.4% (41/53) of patients, the neutrophil clone was larger than the monocyte clone. Overall, clone sizes in neutrophils closely resembled those in monocytes, whereas lymphocyte clones showed greater variability. Based on these findings, clonal dynamics over time were analyzed independently of treatment, confirming a strong correlation between neutrophil and monocyte clone sizes.

Initial clone sizes ≥ 90% were observed in 24.5% (13/53) of patients for GPI‐deficient neutrophil and in 18.9% (10/53) for monocytes. Initial clone sizes ≥ 50% in neutrophil were found in 60.4% (32/53) of patients, compared to 54.7% (29/53) in monocytes. In 18.9% (10/53), initial clone sizes were ≤ 10% in both neutrophil and monocytes. Among lymphocytes, clone sizes ≥ 10% were detected in only 20.8% (11/53) of patients.

Clonal persistence, defined as a change in clone size of no more than ±10% over time, was observed in the majority of patients across different treatment regimens. Persistence in both neutrophil and monocytes was observed in 45.3% (24/53), while in lymphocytes, it was found in only 22.6% (12/53).

An increase in clone size ≥ 10% was seen in 52.8% (28/53) of patients for lymphocytes, compared to 30.2% (16/53) in neutrophil and 28.3% (15/53) in monocytes. A decrease in clone size > 10% was similarly distributed across all three cell lineages. Clonal loss was observed in 7.5% of patients (4/53), all of whom had undergone stem cell transplantation (Figure 2).

**FIGURE 2:**
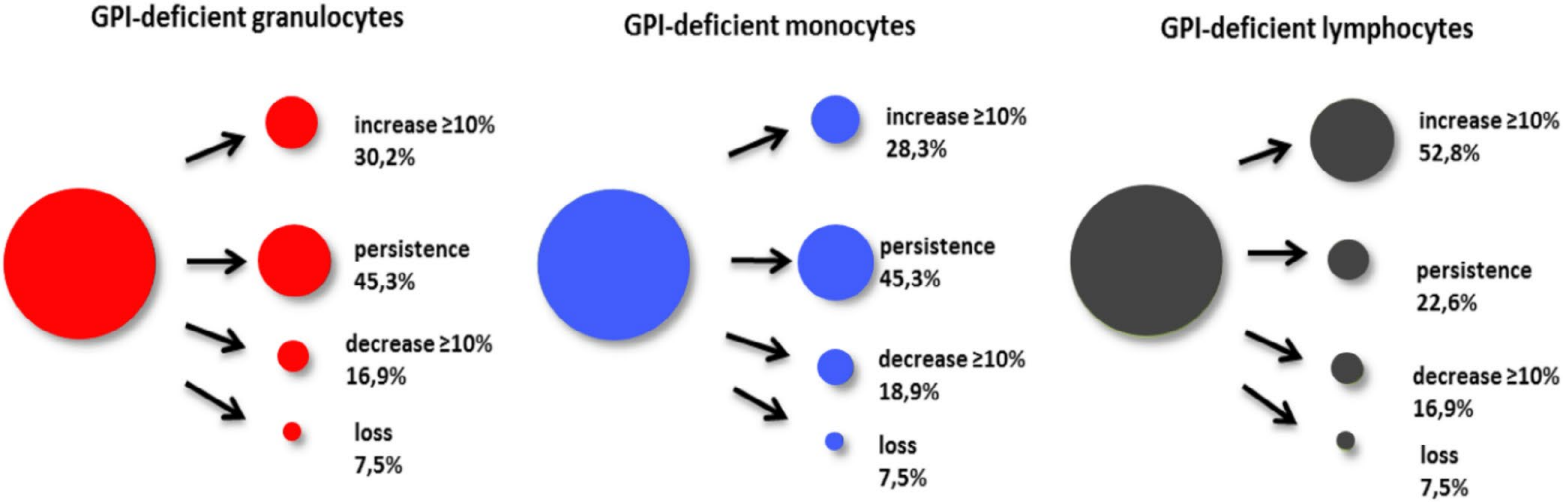
Clonal dynamics of GPI‐deficient granulocytes, monocytes, and lymphocytes over time (*n* = 53). Red dots granulocytes. Blue dots monocytes. Grey dots lymphocytes.

In patients with an initial PNH neutrophil clone size ≥ 90% (13/53), clonal persistence was observed in all cases, while 53.8% (7/13) showed a > 10% increase in lymphocyte clone size over time. Among those with an initial neutrophil clone size between ≥ 50% and < 90%, an increase > 10% was observed in 42.1% (8/19), and clonal persistence in 47.3% (9/19). In contrast, patients with an initial neutrophil clone size < 50% showed a > 10% increase and decrease in 38.1% (8/21) of cases each, indicating a symmetrical pattern of clonal expansion and regression over time. Clonal persistence in this group was observed in only 9.5% (2/21).

Clonal dynamics in GPI‐deficient neutrophil showed a close relationship to clonal dynamics in GPI‐deficient monocytes. In patients with an initial PNH neutrophil clone size ≥ 50% (*n* = 32), clonal persistence was observed in the majority of cases [68.8% (22/32)], while a decrease > 10% in clone size was found only in one patient. In comparison, among patients with an initial GPI‐deficient neutrophil clone size < 50%, we observed a similar distribution of 38.1% (8/21) between clonal decrease and increase over the observation period, while clonal persistence of GPI‐deficient neutrophil was found in only 9.5% (2/21) of these patients.

In 18 of the 53 patients (24.5%) in whom clonal dynamics were assessed across neutrophils, monocytes, and lymphocytes, a divergence in clonal evolution among the three lineages was observed during the study period (Figure 3). This divergence was most pronounced in lymphocytes, whereas clone dynamics in neutrophils and monocytes were strongly correlated, with the exception of five patients.

**FIGURE 3:**
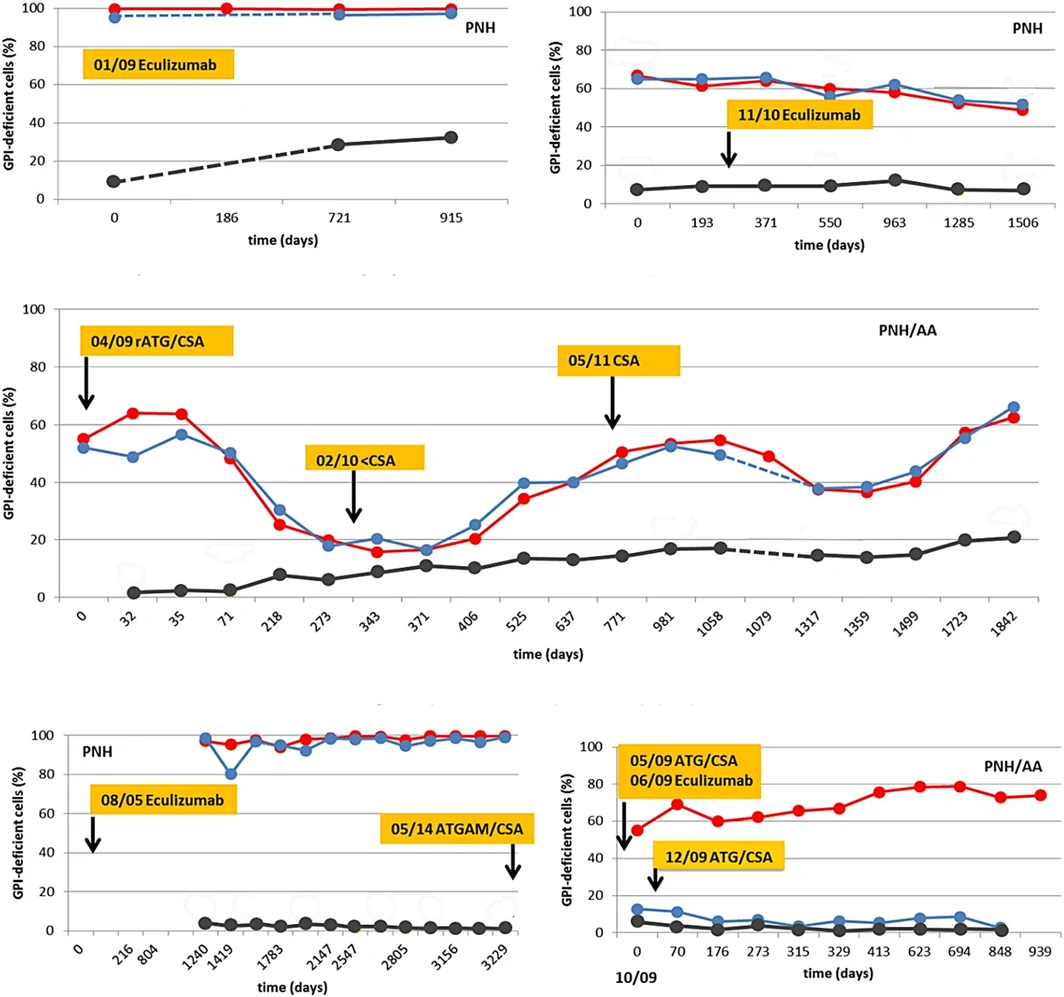
Examples of divergent clonal dynamics over time (*n* = 5). Red dots granulocytes. Blue dots monocytes. Grey dots lymphocytes.

Overall, clonal expansion of GPI‐deficient neutrophils ≥ 10% over time was observed in 24 of the evaluated patients (Figure 4). The median observation time in this group was 34.4 months (range 2.6–114.6 months). In these patients, the initial median PNH clone size was 38.9% (range 0.42%–88.7%), increasing to 71.9% (range 1.2%–99.8%) at final follow‐up. In 12 of the 24 patients (50%), the initial clone size was < 50%.

**FIGURE 4:**
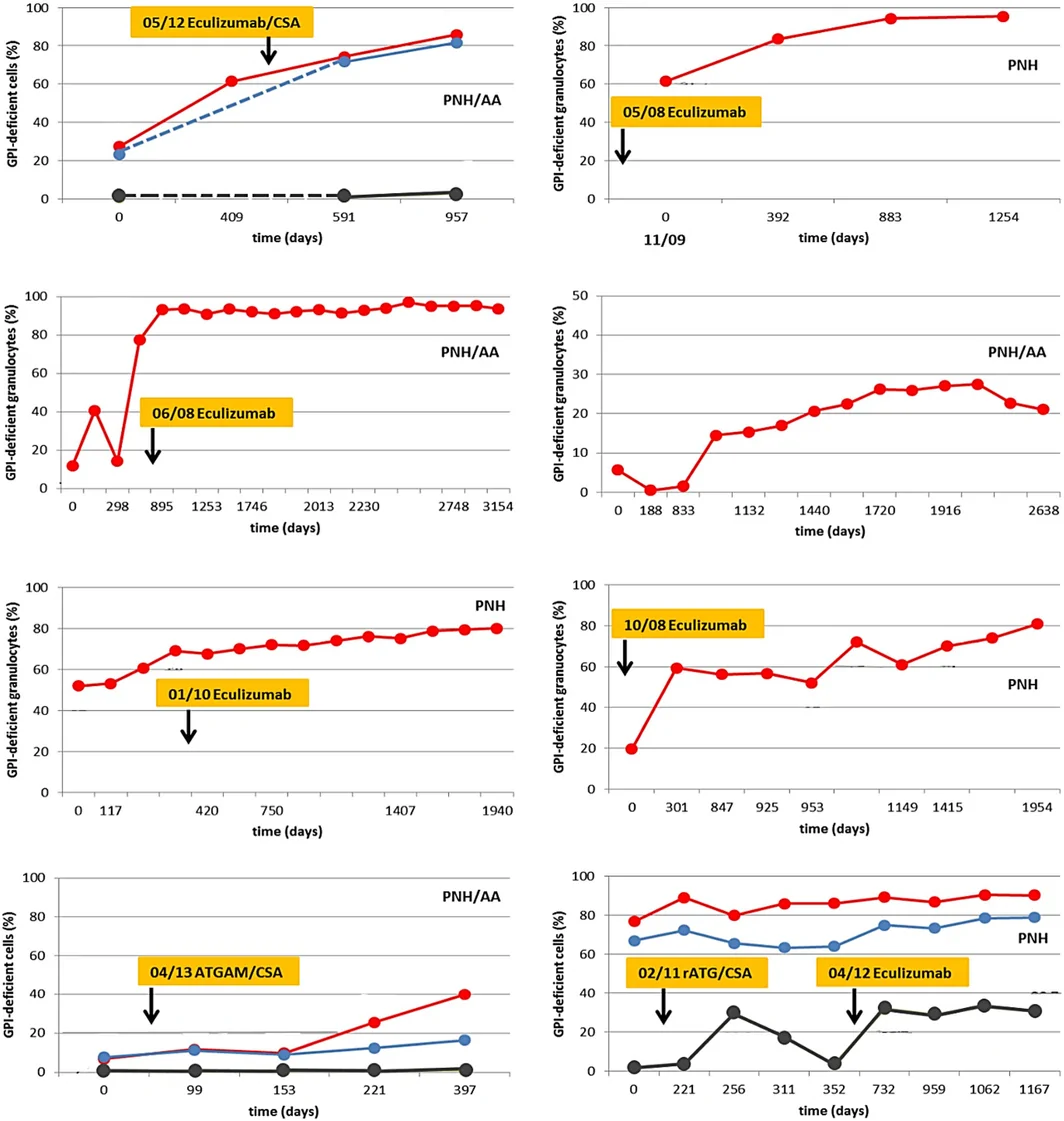
Examples of clonal expansion in PNH‐granulocyte clone size ≥ 10% over time (*n* = 8). Red dots granulocytes. Blue dots monocytes. Grey dots lymphocytes.

In the majority of patients (32/83), clonal persistence was observed over time. The initial median clone size of GPI‐deficient neutrophils in this group was 93.7% (range 0.2%–99.7%), compared to 95.5% (range 0.2%–99.8%) at final follow‐up. In 62.5% of these patients (20/32), the initial neutrophil clone size was ≥ 90%, whereas 9.4% (3/32) had an initial clone size < 50%. The median observation time for patients with persistent neutrophil clones was 27.3 months (range 1.3–108.5 months).

Clonal regression of GPI‐deficient neutrophils ≥ 10% compared to the initial clone size was observed in 13 patients (Figure 5). The initial median clone size in this group was 25.2% (range 7%–78.9%), decreasing to 6.8% (range 1.4%–64.7%) at final follow‐up. The median observation time in this group was 11.2 months (range 3.7–158.9 months). In 76.9% of these patients (10/13), the initial neutrophil clone size was < 50%.

**FIGURE 5:**
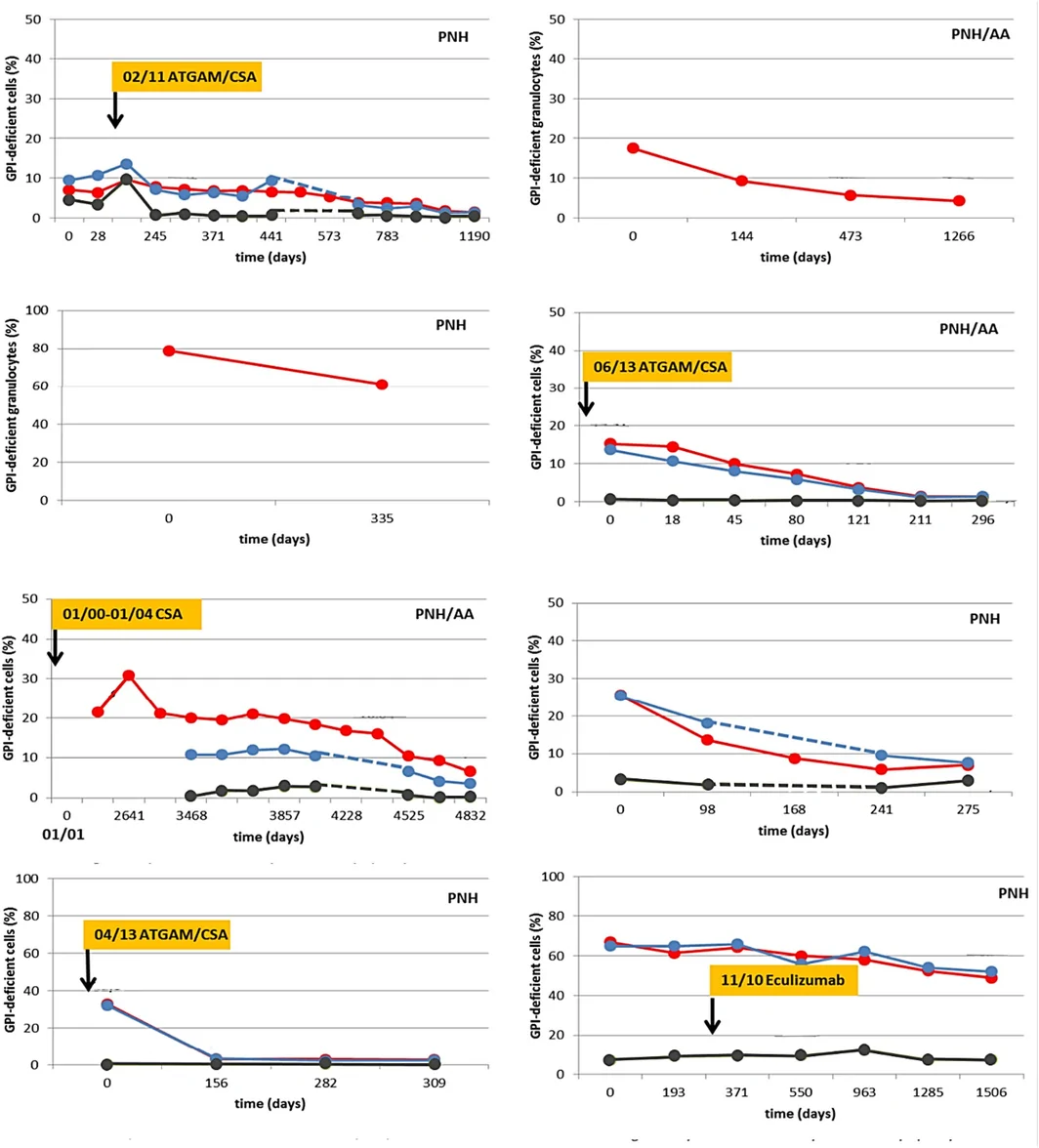
Examples of clonal regression in granulocyte PNH‐clone size ≥ 10% over time (*n* = 8). Red dots granulocytes. Blue dots monocytes. Grey dots lymphocytes.

A spontaneous reduction and eventual disappearance of the detectable neutrophil clone was observed in one patient with AA/PNH, with an initial clone size < 10% (Figure 6).

**FIGURE 6:**
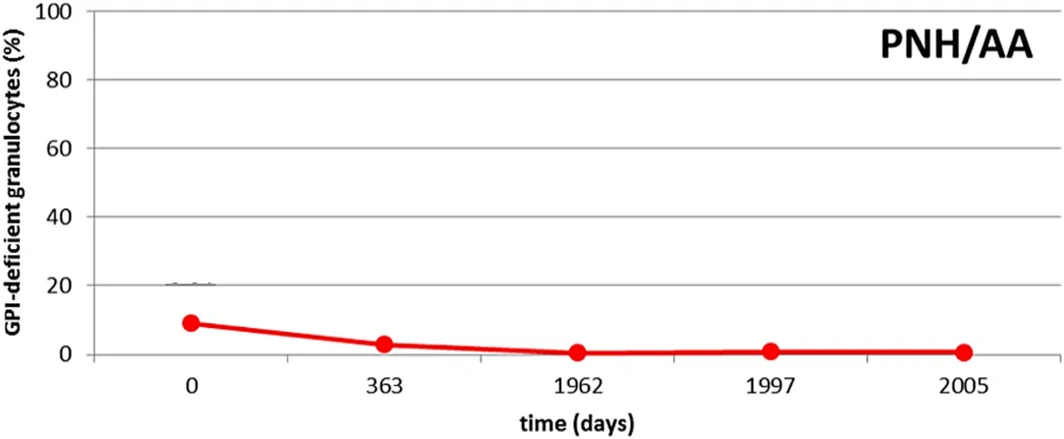
Spontaneous clonal remission in one patient with PNH/AA‐syndrome. Red dots granulocytes.

Among the 108 patients diagnosed and treated at our center, 10 patients with PNH or AA/PNH underwent allogeneic HSCT. In six of these patients, clonal dynamics of GPI‐deficient cells were continuously monitored throughout the observation period. The indication for transplantation was determined by the underlying clinical condition and followed established treatment recommendations. Effective clonal elimination in all three cell lineages was observed in patients following allogeneic HSCT (Figure S1). Two patients died due to transplant‐related complications.

## 4 Discussion

This single‐center retrospective study was designed to provide an intensive overview of clonal dynamics over time in patients with PNH, particularly in the era of complement inhibition. As expected, no consistent effect of eculizumab treatment on white blood cell PNH clone size was observed, since complement inhibition primarily prevents complement‐mediated hemolysis and is not expected to directly affect the underlying hematopoietic clone. Nishimura et al. reported similar distributions of clonal behavior in the pre‐eculizumab era, indicating that complement inhibition does not affect intrinsic hematopoietic stem cell dynamics [17]. These observations reinforce the notion that eculizumab mitigates complement‐mediated hemolysis without influencing the underlying clonal architecture.

Regarding evolution in patients with PNH, the proportions of GPI‐deficient neutrophils and monocytes are generally considered robust indicators of the underlying PNH clone size [18, 19, 20, 21]. PNH clone size on neutrophils is not affected by red cell transfusion and the lifespan of PNH neutrophils is normal compared to GPI‐deficient erythrocytes, which are susceptible to complement‐mediated intravascular hemolysis [18, 19, 20, 21]. The size of the PNH clone was reported as the highest value among PNH clones on neutrophils, monocytes, or erythroid cells, while in some patients with classic PNH, the monocyte clone was often the largest [10, 22]. For the evaluation of white blood cells, the analysis of neutrophils is advisable and the findings should be confirmed by analysis of monocytes. Moreover, in patients with severe neutropenia, testing of monocytes, rather than neutrophils, is particularly useful in estimating true clone size. In contrast to the expression of GPI‐linked proteins on neutrophils and monocytes, gating of lymphocytes may be useful as an internal reference population, although their role in reflecting clonal dynamics remains limited, as the prevalence of GPI‐negative cells may be underestimated.

Among the 108 patients diagnosed and treated at our center, clonal evolution on neutrophils was continuously monitored using FLAER over time in 83 patients with either PNH or PNH/AA syndrome. Median observation time was 29.4 months (range 1.1–162.7 months), and treatment in these patients followed current consensus guidelines, including eculizumab, ATG in combination with cyclosporine (CsA), and HSCT.

In other related studies conducted in the past, three main outcomes have been described: clonal stability, expansion, or regression, which is consistent with the findings of the present study [23, 24, 25]. Our findings are generally consistent with previous longitudinal studies reporting clonal persistence, expansion, and regression in PNH patients over time [26, 27].

Overall, the initial median clone size of GPI‐deficient neutrophils among the 83 monitored patients was 62.7% (range 0.1%–99.7%) compared to 70.9% (range 0%–99.8%) on the primary endpoint of the present evaluation. In the majority of our patients with PNH or PNH/AA syndrome (32/83), clonal persistence throughout the observation period was observed regarding clone size in GPI‐deficient neutrophils. Median observation time in this group was 27.3 months (range 1.3–108.5 months). In 20 out of these 32 patients, the initial clone size in GPI‐deficient neutrophils, defined as the first measured clone size by FLAER in our outpatient clinic, was ≥ 90%. One possible explanation for this finding could be that in the majority of patients, the process of autoimmune selection or the acquisition of further mutations [28, 29, 30] required for clonal expansion had already resolved by the time of diagnosis. A divergent clonal evolution was seen in 12 out of the 32 patients and was mainly related to PNH clones in GPI‐deficient lymphocytes, showing clonal expansion in nine patients over time. In eight of these nine patients, the initial clone size of GPI‐deficient lymphocytes was ≤ 10%. This observation may suggest that the proportion of GPI‐deficient lymphocytes is thought to correlate with the actual disease duration, with larger clones suggesting a longer‐standing course, as PNH‐lymphocytes may representing a more stable compartment compared to neutrophils and erythrocytes. However, this observation should be interpreted with caution. The lymphocyte compartment is heterogeneous and comprises T cells, B cells, and NK cells with substantially different life spans and turnover kinetics. In addition, age, thymic output, aplastic marrow failure, immune reconstitution, and immunosuppressive therapy may influence lymphocyte composition and clonal representation. Therefore, lymphocyte PNH clone size is not directly comparable to neutrophil or monocyte clone size and should not be considered a primary parameter for routine clinical monitoring [10, 11, 15, 16, 31].

Expansion of the PNH cell population on neutrophils was seen in 24 patients. The initial median clone size of GPI‐deficient neutrophils in this group was 38.9% (range 0.4%–88.7%) compared to 71.9% (range 1.2%–99.8%) at the final day of observation. Median observation time was 34.4 months (range 2.6–114.6 months). At presentation, initial PNH clone sizes in these patients varied widely. In most patients (12/24), the initial clone size was > 50%, compared to ≤ 10% (6/24) or > 10 to ≤ 50% (6/24). In cases of clonal expansion, increases were observed across all three cell lineages, supporting the idea that selective pressure was still ongoing at the time of presentation in our outpatient clinic [28].

In 13 out of the 83 patients (15.7%), there was evidence of clonal regression. Clonal regression was defined as a decrease in the PNH clone size of GPI‐deficient neutrophils and monocytes ≥ 10% compared to the initial value. The initial clone size was defined as the first measurement of PNH clone size by FLAER in our outpatient clinic. In these patients, the initial median clone size of GPI‐deficient neutrophils was 25.2% (range 7%–78.9%), compared to 6.8% (range 1.4%–64.7%) at the final day of observation. Accordingly, in the evaluated PNH and AA/PNH patients with small PNH clones at baseline, clonal expansion over time was absent, despite a theoretical capacity for growth. Thus, clonal regression was still ongoing in these patients and had not yet plateaued. These findings support the observation that clonal regression is more likely to occur in patients with small PNH clones at diagnosis. In our cohort, one patient experienced spontaneous reduction and eventual disappearance of the detectable neutrophil clone during follow‐up (1 of 83; 1.2%). Similar observations were reported by Ferla et al. (3 of 55 patients; 5.5%) with clinical remission [32], and Gurnari et al. (3 of 92 patients; 3.3%) with molecular remission [33]. Although the reported rates vary somewhat across studies, they consistently indicate that spontaneous remissions occur in approximately 1%–6% of PNH patients [32, 33]. This concordance underscores that spontaneous remission, although uncommon and rare, is a reproducible phenomenon that warrants further investigation into the underlying mechanisms. Of note, in such states, GPI‐deficient lymphocytes may persist despite the disappearance of affected neutrophils, monocytes, and erythrocytes. This supports the concept that lymphocyte clones may represent a residual “scar” or remnant of the original PNH clone. Accordingly, neutrophil and monocyte PNH clone sizes remain the most clinically relevant parameters for routine monitoring, whereas lymphocyte analysis may primarily provide exploratory or pathophysiological information in selected longitudinal studies.

Depending on patient age and the availability of a suitable human leukocyte antigen (HLA)‐matched donor, HSCT remains the only curative treatment option in patients with SAA, with or without a PNH clone [34, 35]. In patients with PNH, HSCT is generally postponed until disease progression manifests as recurrent life‐threatening thromboembolism, refractory or transfusion‐dependent hemolytic anemia, or development of SAA [31, 36]. Although HSCT enables effective clonal elimination, the risk of treatment‐related mortality remains high, particularly due to graft‐versus‐host disease (GvHD) [37]. Ten patients treated at our center with either PNH or PNH/AA syndrome underwent HSCT. Two of these patients died due to treatment‐related mortality. In six patients, clonal dynamics were continuously monitored following HSCT, and complete eradication of PNH cells was observed in all, supporting data from previous multicenter studies regarding the efficacy of HSCT in eliminating PNH clones (Figure S1).

Consistent with previous studies, eculizumab treatment was not associated with relevant changes in white blood cell PNH clone size, supporting the concept that complement inhibition does not directly influence clonal hematopoiesis. In contrast to other related studies [28], in six patients treated with ATG plus CsA, a clonal regression over time could be observed. In these ATG‐treated patients initial PNH population was ≤ 50%.

Routine disease monitoring by flow cytometry is recommended for all PNH patients, in accordance with current guidelines [31, 38]. The present investigation reveals considerable variability in the kinetics of clone development. Future studies should focus on the relationship between these kinetics and the clinical outcome.

## 5 Limitations

This study has several limitations. First, its retrospective single‐center design may introduce selection bias. Second, the cohort was clinically heterogeneous and included patients with classic PNH as well as AA/PNH receiving different treatment modalities. Third, follow‐up intervals were not standardized and reflected routine clinical practice. Finally, interpretation of very small PNH clones requires caution, as analytical and biological variability may have a greater impact in this setting. Despite these limitations, the study provides longitudinal real‐world data on PNH clone dynamics across different cell populations.

## 6 Conclusion

The present follow‐up study refines our understanding of PNH clone dynamics. Clone size in neutrophils mirrors that in monocytes, whereas lymphocyte clones frequently diverge; dual‐lineage assessment is therefore essential, particularly in neutropenic patients, to avoid underestimating the true disease burden. In addition, GPI‐deficient lymphocytes may provide exploratory information regarding long‐term clonal persistence, although their clinical significance requires further investigation. Most clones remained stable, yet a sizeable minority expanded, and expansion and regression were similarly frequent when the initial neutrophil clone was < 50%. These trajectories proved independent of C5 inhibition with eculizumab and of ATG–cyclosporine immunosuppression, and we documented both a complete spontaneous remission and uniform clone eradication after allogeneic transplantation.

Contrasting an earlier Asian cohort that analyzed only the granulocyte–erythrocyte axis [33], our FLAER‐based European series, conducted in the complement‐blockade era, demonstrates that neutrophil‐plus‐monocyte monitoring provides the most reliable gauge of clonal dynamics. Taken together, the data support routine longitudinal measurement of these two lineages to guide clinical decisions and highlight the need for further studies to define the molecular and immunologic forces governing clonal expansion, persistence, and regression in paroxysmal nocturnal hemoglobinuria.

## Funding

The authors have nothing to report.

## Ethics Statement

This study was performed in accordance with the study protocol, the Declaration of Helsinki, and the International Council for Harmonization guidelines for Good Clinical Practice. All patients provided written informed consent for their participation in the study.

## Conflicts of Interest

The following authors S.M.F., F.P., U.S., and N.P. declares no conflicts of interest. H.C.R. reports consultancy for Roche, Vertex, Janssen, and KinSea; honoraria from Abbvie, Aurikamed, Roche, Novartis, Takeda, and Amgen; research funding from Gilead and AstraZeneca; ownership interests in Fresenius, Roche, Novartis, Lilly, and Janssen; and is founder of CDL Therapeutics GmbH. A.R. reports consultancy fees from Alexion Pharmaceuticals Inc., Apellis Pharmaceuticals, Bioverativ, a Sanofi company, Novartis, Roche, Recordati and Sanofi; honoraria from Alexion, Amgen, Apellis, Novartis, Roche, Sanofi and Sobi, and advisory board fees from Alexion, Amgen, Apellis, Bioverativ, Novartis, Roche, Recordati, Sanofi, and Sobi. F.A. reports consultancy honorarium for participation in advisory boards from Novartis, Bristol Myers Squibb, Global Blood Therapeutics/Pfizer, Vertex and Avanzanite/Agios, and received a honorarium from Avanzanite/Agios, Novartis, Bristol Meyers Squibb, Global Blood Therapeutics/Pfizer and Vertex for consultancy.

## Supporting information


**Figure S1:** Effective PNH‐clone elimination following allogeneic hematopoietic stem cell transplantation (*n* = 4). Red dots granulocytes. Blue dots monocytes. Grey dots lymphocytes.

## Data Availability

The data that support the findings of this study are available on request to the corresponding author.
