# Supplementary material for: Clonal Dynamics of GPI‐Deficient Cells in Patients With Paroxysmal Nocturnal Hemoglobinuria (PNH): A Retrospective Follow‐Up Analysis
Source: Eur J Haematol. 2026 Aug 21;117(6):1583–92. doi: 10.1111/ejh.70293 (PMC13729962; doi:10.1111/ejh.70293)
Supplement: Supplementary file 1 [file EJH-117-1583-s001.docx]

**Clonal Dynamics - Supplement Figures and Legends**

SUPPLEMENTARY FIGURE 1.

**Figure legend**

SUPPLEMENTARY FIGURE 1. Effective PNH-clone elimination following allogeneic hematopoietic stem cell transplantation. (n=4). Red dots granulocytes. Blue dots monocytes. Grey dots lymphocytes.
